# Supplementary material for: Development and evaluation of a stepwise clinical competency development program for new nurses: A single-group repeated-measures quasi-experimental study
Source: PLoS One. 2026 Feb 9;21(2):e0342464. doi: 10.1371/journal.pone.0342464 (PMC12885250; doi:10.1371/journal.pone.0342464)
Supplement: Attachment — Submitted filename: Response to Reviewer #2.docx [file pone.0342464.s003.docx]

**Dear Reviewer,**

Thank you for your insightful feedback on our manuscript. We greatly value your thorough review and have made the necessary revisions in response to your comments. We hope that these adjustments meet your expectations.

| **Comment** | **Revision** | |
| --- | --- | --- |
|  | **Before** | **After** |
| Overall, a well developed detailed manuscripts. The theme of teh research shows relevancy to the readers and need of the time. I myself being a nurse educator totally agree to implementation of bootcamps, trainings and orientations for novice nurses and allied health. However, there is a clarity required as to 3 questionnaires of clinical knowledge, performance and self efficacy were used all together before, immediate and after training. The training modules doesnot have ECG machine, Cardiac monitor, Pharmacology, leadership, critical thinking, basic nursing skills such as Nasogastric insertion, feeding, catheterization topics in it. Training modules need sequence may b elater on, It will be effective to use controlled and uncontrolled group methodology to see effectiveness of the training perhaps later on stages it can be developed. Does participants got exhausted with lengthy questionnaires, it could be a limitation. | **Program Development**  The program was divided into two stages, basic and advanced, with each stage structured around clearly defined competencies required for new nurses to progress from beginner to practitioner levels (Table 1). Each SCCDP session was developed using theoretical content, practice, and simulation-based education methods to accommodate diverse learning styles and facilitate the evaluation of practical competencies (Figure. 2). The SCCDP provides systematic and comprehensive education that enables new nurses to gradually develop clinical competencies through formal education and preceptorship support over time. During their first eight weeks of employment, new nurses participated in the program’s basic phase alongside a preceptorship course. This phase included four hours of formal training sessions held every two weeks. The basic level covered foundational clinical skills and knowledge, such as drug administration, proper use of medical devices, and infection control. Additionally, it comprehensively addressed essential competencies for the hospital environment, including Situation, Background, Assessment, Recommendation (SBAR)-based handover training, communication, and teamwork. Four months after joining the hospital, new nurses transitioned to the advanced stage of SCCDP. This stage consisted of four sessions, each lasting 8 hours and held at 2-month intervals, covering emergency nursing, respiratory system nursing, renal system nursing, and circulatory system nursing. The advanced stage emphasized enhancing decision-making and critical thinking skills in complex clinical situations by integrating theoretical education and clinical scenario-based simulations. A key feature of this stage was the provision of immediate and specific feedback following each session to support learners’ continuous development. Throughout the program period, an online platform called Padlet was used, allowing new nurses to ask questions in real time about the challenges encountered in practice, with the training nurse providing immediate feedback. This allowed new nurses to receive continuous support regardless of time and location.  Additionally, to address the individual needs of learners, regular interviews were conducted among new nurses, preceptors, and the clinical nurse educator, with additional support provided as necessary. For new nurses who were slower to learn or adapt to their roles, a dedicated training nurse provided one-on-one shadowing sessions at a clinical site. This individualized support aimed to address the unique needs of each learner, ensuring that all new nurses developed the required competencies. To encourage self-directed learning, clear learning objectives and expected competencies were outlined at the beginning of each stage, and self-assessment tools were provided to help learners track their progress. Through this approach, learners were able to recognize their strengths and areas for improvement, and actively engaging in their learning process. To continually assess and enhance the program’s effectiveness, regular feedback was gathered from new nurses and preceptors, allowing for ongoing revisions to the program's content and structure. Expert validation of the SCCDP, conducted by three nursing school professors, two clinical nurse educators, and two nursing managers with master's degrees or higher, resulted in a content validity index (CVI) of .95. Table 1 provides detailed information about the content covered in each session.  **DISCUSSION**  This study developed and implemented an SCCDP to improve the clinical performance of new nurses, support their effective adaptation to clinical settings, and increase their intention to remain in the workplace. To assess its effectiveness, the SCCDP was structured into basic and advanced stages and applied over time. Clinical knowledge, clinical performance ability, and self-efficacy were evaluated to confirm the program’s effectiveness. Verification of the effectiveness of the SCCDP revealed improvements in both basic and advanced clinical knowledge and clinical performance ability following training. Notably, the SCCDP demonstrated a lasting impact, with effects sustained immediately after implementation and continuing at three and six months post-training. This is more thoroughly discussed below.  …….  This study proposes an effective program to strengthen the clinical competency and practice adaptation of new nurses. However, it has several limitations. First, additional educational interventions are required to further develop SBAR and handover skills. Second, educational strategies that incorporate long-term mentoring and continuous feedback are necessary to enhance psychological competencies such as self-efficacy. Third, since our study used a convenient sample from a single medical institution, caution must be exercised when generalizing the findings. In the future, the program should be expanded and applied across diverse hospitals and clinical settings and continuously improved through feedback. | **Program Development**  The program was divided into two stages, basic and advanced, with each stage structured around clearly defined competencies required for new nurses to progress from beginner to practitioner levels (Table 1). Each SCCDP session was developed using theoretical content, practice, and simulation-based education methods to accommodate diverse learning styles and facilitate the evaluation of practical competencies (Fig 2). The lectures and practical sessions were delivered by two clinical nurse educators with more than seven years of experience, supported by five assistant educators. Feedback on individual learning needs was provided by the same educators. A total of seven educators were involved in the delivery and evaluation of the program to ensure consistency and minimize potential bias or errors. The SCCDP provides systematic and comprehensive education that enables new nurses to gradually develop clinical competencies through formal education and preceptorship support over time. During their first eight weeks of employment, new nurses participated in the program’s basic phase alongside a preceptorship course. This phase included four hours of formal training sessions held every two weeks. The basic level covered foundational clinical skills and knowledge, such as drug administration, proper use of medical devices, and infection control. Additionally, it comprehensively addressed essential competencies for the hospital environment, including Situation, Background, Assessment, Recommendation (SBAR)-based handover training, communication, and teamwork. It is worth noting that basic nursing procedures such as nasogastric tube insertion, feeding, and urinary catheterization were not included in the SCCDP modules, as these skills are typically taught and supervised by preceptor nurses within the clinical departments during routine patient care.  Four months after joining the hospital, new nurses transitioned to the advanced stage of SCCDP. This stage consisted of four sessions, each lasting 8 hours and held at 2-month intervals, covering emergency nursing, respiratory system nursing, renal system nursing, and circulatory system nursing. The advanced stage emphasized enhancing decision-making and critical thinking skills in complex clinical situations by integrating theoretical education and clinical scenario-based simulations. A key feature of this stage was the provision of immediate and specific feedback following each session to support learners’ continuous development. Practical components in this stage were implemented using action learning strategies, which are known to support the development of leadership and critical thinking competencies through team-based problem-solving and reflection. Throughout the program period, an online platform called Padlet was used—a real-time, web-based interactive bulletin board that facilitates asynchronous communication and content sharing among participants and educators [https://padlet.com]. This enabled new nurses to ask questions anytime and receive prompt feedback from the training nurse. This phase incorporated hands-on sessions grounded in action learning strategies, facilitating collaborative decision-making among participants and fostering the development of critical thinking and effective leadership competencies essential for nursing professionals through reflective, experiential, and practice-based learning approaches [19].  Additionally, to address the individual needs of learners, regular interviews were conducted among new nurses, preceptors, and the clinical nurse educator, with additional support provided as necessary. For new nurses who were slower to learn or adapt to their roles, a dedicated training nurse provided one-on-one shadowing sessions at a clinical site. This individualized support aimed to address the unique needs of each learner, ensuring that all new nurses developed the required competencies. To encourage self-directed learning, clear learning objectives and expected competencies were outlined at the beginning of each stage, and self-assessment tools were provided to help learners track their progress. Through this approach, learners were able to recognize their strengths and areas for improvement, and actively engaging in their learning process. To continually assess and enhance the program’s effectiveness, regular feedback was gathered from new nurses and preceptors, allowing for ongoing revisions to the program's content and structure. Expert validation of the SCCDP, conducted by three nursing school professors, two clinical nurse educators, and two nursing managers with master's degrees or higher, resulted in a content validity index (CVI) of .95. Table 1 provides detailed information about the content covered in each session.  **Discussion**  This study developed and implemented an SCCDP to improve the clinical performance of new nurses, support their effective adaptation to clinical settings, and increase their intention to remain in the workplace. To assess its effectiveness, the SCCDP was structured into basic and advanced stages and applied over time. Clinical knowledge, clinical performance ability, and self-efficacy were evaluated to confirm the program’s effectiveness. Verification of the effectiveness of the SCCDP revealed improvements in both basic and advanced clinical knowledge and clinical performance ability following training. Notably, the SCCDP demonstrated a lasting impact, with effects sustained immediately after implementation and continuing at three and six months post-training. This is more thoroughly discussed below.  …….  This study proposes an effective program to strengthen the clinical competency and practice adaptation of new nurses. However, it has several limitations. First, additional educational interventions are required to further develop SBAR and handover skills. Second, educational strategies that incorporate long-term mentoring and continuous feedback are necessary to enhance psychological competencies such as self-efficacy. Third, since our study used a convenient sample from a single medical institution, caution must be exercised when generalizing the findings. Additionally, as all three instruments—clinical knowledge, clinical performance ability, and self-efficacy—were administered simultaneously at each measurement point, participants may have experienced fatigue due to the length of the questionnaires. This potential burden may have influenced the accuracy or completeness of responses, and should be considered a limitation of the study. Additionally, the absence of a control group limits the ability to determine causality in observed improvements. Future studies should consider employing controlled or randomized trial designs to more rigorously evaluate the program's effectiveness. In the future, the program should be expanded and applied across diverse hospitals and clinical settings and continuously improved through feedback. |
