# Supplementary material for: Development and evaluation of a stepwise clinical competency development program for new nurses: A single-group repeated-measures quasi-experimental study
Source: PLoS One. 2026 Feb 9;21(2):e0342464. doi: 10.1371/journal.pone.0342464 (PMC12885250; doi:10.1371/journal.pone.0342464)
Supplement: Attachment — Submitted filename: Plos One Manuscript .docx [file pone.0342464.s002.docx]

**Development and evaluation of a stepwise clinical competency development program for new nurses: A single-group repeated-measures quasi-experimental study**

Shin Hye Ahn^1^, Hye Won Jeong^2*^

^1^ Department of Nursing, Sunchon National University, 255, Jungang-ro, Suncheon-si, Jeollanam-do, 57922, Republic of Korea

^2^ Department of Nursing, Korea National University of Transportation, 61, Daehak-ro, Yonggang-ri, Jeungpyeong-eup, Jeungpyeong-gun, Chungcheongbuk-do, 27909, Republic of Korea

*Corresponding author

E-mail: [hyewon1129@ut.ac.kr](mailto:hyewon1129@ut.ac.kr)

**Abstract**

Background: New nurses often encounter adaptation challenges in hospital settings owing to gaps in clinical knowledge and skills, which contributes to high turnover rates, affects hospital efficiency, and increases the burden on healthcare teams. Effective training is essential to support clinical adaptation. This study aimed to develop a structured, stepwise clinical competency development program (SCCDP) for new nurses and evaluate its effectiveness. Methods: This study employed a single-group repeated-measures quasi-experimental design. From September 2023 to July 2024, 49 newly recruited nurses from C University Hospital in South Korea, participated in the SCCDP. The program was divided into basic and advanced stages and incorporated theoretical content, practice, and simulation. The outcomes measured included basic and advanced clinical knowledge, clinical performance ability, and self-efficacy at four intervals: pre-intervention, immediately post-intervention, and at three and six months post intervention . Results: The SCCDP significantly improved new nurses’ basic clinical knowledge immediately post-intervention and three and six months post-completion (F = 40.01, *p* < .001), with the largest effect size observed in medical device operation (ES = 1.56 – 1.65). Advanced clinical knowledge also demonstrated significant enhancement across all time points (F = 26.06, *p* < .001), with the greatest increase occurring immediately after the program (ES = 1.84), particularly in emergency nursing (ES = 1.22). Clinical performance ability showed notable gains at three and six months post-program (χ² = 55.92, *p* < .001), with the most substantial improvement in interpersonal and communication skills (ES = 2.05 – 2.09). However, self-efficacy did not change significantly over time (F = 2.80, *p* = .066). Conclusions: The SCCDP effectively enhanced new nurses’ clinical knowledge and performance abilities, supporting their adaptation to the clinical environment. However, self-efficacy did not significantly improve, suggesting the need for additional support. The program shows promise in promoting clinical adaptation and reducing turnover among newly recruited nurses.

**Keywords:** Nursing education, Competency-based education, Clinical competence, On the job training, Program evaluation

**Introduction**

The turnover rate among new nurses with less than one year of employment following their 2022 appointments was 52.8%, and this trend continues to rise [1]. New nurses experience challenges in adapting to the hospital’s organizational environment, often due to insufficient knowledge and professional skills, excessive workload, and difficulties in communication and collaboration with other medical staff and colleagues [2]. Nurse turnover leads to significant financial losses for hospitals [3], with new nurse turnover comprising the largest portion of the overall nursing attrition [1], which further increases the burden on patients and remaining staff. To support new nurses in adapting effectively to the clinical setting, organizations must implement strategies that reduce turnover intention and job stress while enhancing self-efficacy [4].

To address the limitations of preceptorship and the previous education system for new nurses and to support new nurses’ adaption to clinical practice, there has been a call to implement an effective educational system and deploy dedicated training personnel [5]. The role of clinical nurse educators is gaining prevalence worldwide, as they provide quality education and support new nurses in their transition into confident, competent practitioners [6]. To establish a systematic, practice-oriented curriculum and training staff that reflect the unique characteristics of the working environment and healthcare institutions [7], the Ministry of Health and Welfare and the Korean Nurses Association launched a support initiative in 2019 for clinical nurse educators and training programs for new nurses, accompanied by the release of management system guidelines [8]. Clinical nurse educators, primarily deployed at national and public hospitals, provide essential job training, identify the clinical difficulties experienced by new nurses, develop practical educational programs, and deliver standardized training for complex nursing skills [8]. However, the current circumstances of Korean medical institutions, which include a widely varied training period and curriculum for new nurses across institutions and insufficient deployment of clinical nurse educators, limited their ability to fully support new nurses in adapting to the clinical environment [7, 9].

Among the various factors influencing turnover rates perceived by new nurses, work intensity was identified as the most significant, followed by practical competency, which accounted for approximately 20.5% [10]. Insufficient preparation for practice can lead to transition shock, underscoring the need for clinical nursing education that supports the transition process in clinical settings [11]. Education and effective training that foster critical thinking skills are essential for enhancing the clinical practice competency of new nurses [12]. Providing recurring experiences closely aligns with clinical practice, along with education and constructive feedback tailored to the competency levels of new nurses, which has been shown to improve their practical skills [11]. To strengthen new nurses’ practical skills, support their adaptation to the clinical setting, and reduce turnover associated with insufficient competency, it is necessary to develop and implement a practical competency enhancement program for them led by dedicated clinical nurse educators.

As new nurses encounter increasing challenges during their transition from beginners to fully qualified practitioners, a step-by-step education and development strategy must be established that considers their tenure and professional growth processes [13]. Accordingly, this study aimed to develop and implement a stepwise clinical competency development program (SCCDP) tailored to the tenure of new nurses, assess its effectiveness, and provide foundational data for a standardized integrated adaptation support program that can be applied to new nurses in the future. Ultimately, this approach is expected to enhance the quality of patient care and improve job satisfaction by supporting new nurses to strengthen their competencies and adapt to clinical environments [14-16].

Is the problem in the way nurses are trained in your setting? Consider exploring that

What is the implication of these turnover and lack of competences to patient outcomes? Any evidence?

**Conceptual framework**

The SCCDP for new nurses was structured based on the theoretical content of competency-based education (CBE) (Fig 1). CBE is an educational approach focused on ensuring that learners acquire specific competencies and effectively apply them to real-life situations [17]. This conceptual framework is designed to enhance the clinical adaptation and competency of new nurses and incorporates several key elements. At the core of the SCCDP is a step-by-step practical competency-strengthening process that integrates the CBE principles. The SCCDP is divided into basic and advanced stages, each structured around clearly defined competency goals. This approach facilitates the gradual development of competencies among new nurses, enabling flexible education tailored to their learning levels [15].

Competency-based assessment, the core principle of CBE, is another key element of this framework. This evaluation method continuously monitors the learning progress of new nurses and measures their performance in real clinical situations, extending beyond mere knowledge acquisition [16]. Evaluations were conducted before the program, immediately after, and three and six months after to track changes in competency over time. This approach enabled a comprehensive assessment of both the short- and long-term effects of the program. To evaluate the program’s effectiveness, various outcome variables, including basic and advanced clinical knowledge, clinical performance ability, and self-efficacy, were measured. Basic and advanced clinical knowledge evaluated the theoretical foundation of nursing and the understanding of complex clinical situations, whereas clinical performance measured the ability to apply nursing skills in real-world settings. Self-efficacy was used to assess new nurses’ confidence in performing nursing tasks. This multifaceted assessment allowed us to comprehensively measure the overall competency development in new nurses [14]. Continuous feedback, a key component of this framework and an important feature of CBE, helps individuals recognize their strengths and areas for improvement, thus enabling goal-oriented learning [15].

Finally, the framework was designed to be flexible and adaptable. The ability to adjust programs to meet new competency requirements or respond to changes in the healthcare environment is an important feature of the rapidly evolving field of healthcare [15]. This flexibility allows the program to continuously incorporate the latest medical practices and educational methodologies. This conceptual framework aimed to bridge the gap between theory and practice and promote effective clinical adaptation by applying CBE principles to the nursing education of new nurses. The program’s long-term effectiveness can be assessed by tracking changes in competency over time, contributing to its continuous improvement and optimization.

[Insert Fig 1 here]

Materials and methods

Research design

This study employed a single-group, repeated-measures quasi-experimental design to assess the impact of the SCCDP on new nurses’ basic and advanced practical knowledge, clinical performance ability, and self-efficacy.

**Participants**

In this study, new nurses were operationally defined as individuals with less than one year of clinical experience at the hospital or even previous? who were newly employed at a university hospital in Korea between August and December 2023. The inclusion criteria included those who provided consent for this study and completed the entire training. Those who partially completed the program or withdrew from the study were excluded from the analyses. The sample size was calculated using the G*Power 3.1.9.7 software. A medium effect size (f=0.25), based on the effect size reported by Kim et al. [18], was applied with a significance level of .05 set for a single-group repeated measures analysis of variance (ANOVA). With a power of .95, the minimum sample size required was 43. To account for the anticipated dropout rate of approximately 20%, 50 participants were initially recruited. Since one participant dropped out due to resignation, the final sample size was 49.

**Program Development**

The program was divided into two stages, basic and advanced, with each stage structured around clearly defined competencies required for new nurses to progress from beginner to practitioner levels (Table 1). Each SCCDP session was developed using theoretical content, practice, and simulation-based education methods to accommodate diverse learning styles and facilitate the evaluation of practical competencies (Fig 2). Was the training materials used in this study validated? What approach of validation? Who validated? The lectures and practical sessions were delivered by two clinical nurse educators with more than seven years of experience, supported by five assistant educators. Feedback on individual learning needs was provided by the same educators. A total of seven educators were involved in the delivery and evaluation of the program to ensure consistency and minimize potential bias or errors. The SCCDP provides systematic and comprehensive education that enables new nurses to gradually develop clinical competencies through formal education and preceptorship support over time. During their first eight weeks of employment, new nurses participated in the program’s basic phase alongside a preceptorship course. This phase included four hours of formal training sessions held every two weeks. The basic level covered foundational clinical skills and knowledge, such as drug administration, proper use of medical devices, and infection control. Additionally, it comprehensively addressed essential competencies for the hospital environment, including Situation, Background, Assessment, Recommendation (SBAR)-based handover training, communication, and teamwork. It is worth noting that basic nursing procedures such as nasogastric tube insertion, feeding, and urinary catheterization were not included in the SCCDP modules, as these skills are typically taught and supervised by preceptor nurses within the clinical departments during routine patient care.

Four months after joining the hospital, new nurses transitioned to the advanced stage of SCCDP. This stage consisted of four sessions, each lasting 8 hours and held at 2-month intervals, covering emergency nursing, respiratory system nursing, renal system nursing, and circulatory system nursing. The advanced stage emphasized enhancing decision-making and critical thinking skills in complex clinical situations by integrating theoretical education and clinical scenario-based simulations. A key feature of this stage was the provision of immediate and specific feedback following each session to support learners’ continuous development. Practical components in this stage were implemented using action learning strategies, which are known to support the development of leadership and critical thinking competencies through team-based problem-solving and reflection. Throughout the program period, an online platform called Padlet was used—a real-time, web-based interactive bulletin board that facilitates asynchronous communication and content sharing among participants and educators [https://padlet.com]. This enabled new nurses to ask questions anytime and receive prompt feedback from the training nurse. This phase incorporated hands-on sessions grounded in action learning strategies, facilitating collaborative decision-making among participants and fostering the development of critical thinking and effective leadership competencies essential for nursing professionals through reflective, experiential, and practice-based learning approaches [19].

Additionally, to address the individual needs of learners, regular interviews were conducted among new nurses, preceptors, and the clinical nurse educator, with additional support provided as necessary. For new nurses who were slower to learn or adapt to their roles, a dedicated training nurse provided one-on-one shadowing sessions at a clinical site. This individualized support aimed to address the unique needs of each learner, ensuring that all new nurses developed the required competencies. To encourage self-directed learning, clear learning objectives and expected competencies were outlined at the beginning of each stage, and self-assessment tools were provided to help learners track their progress. Through this approach, learners were able to recognize their strengths and areas for improvement, and actively engaging in their learning process. To continually assess and enhance the program’s effectiveness, regular feedback was gathered from new nurses and preceptors, allowing for ongoing revisions to the program's content and structure. Expert validation of the SCCDP, conducted by three nursing school professors, two clinical nurse educators, and two nursing managers with master's degrees or higher, resulted in a content validity index (CVI) of .95. Table 1 provides detailed information about the content covered in each session.

Were the nurses doing this training alongside their official work schedules?

The content below is similar to what is taught in school to nurses. The period from graduation to employment and prior experience may have varied, and this has potential to vary their competencies Was this considered in the inclusion into the programme?

| Table 1. Overview of curriculum content and teaching methods for SCCDP | | | | |
| --- | --- | --- | --- | --- |
| Phase | **Session** | **Content** | **Time (min)** | **Methods** |
| Basic | 1 | Course introduction | 30 | Lecture |
|  |  | Use of electronic medical record system | 120 | Practice |
|  |  | Intradermal injection | 30 | Practice |
|  |  | Subcutaneous injection | 30 | Practice |
|  |  | Insulin preparation | 30 | Practice |
|  | 2 | Intravenous injection | 60 | Practice |
|  |  | Safe medication nursing | 40 | Lecture |
|  |  | Medication dosage calculation (Basic) | 40 | Lecture & Practical |
|  |  | Medication nursing simulation | 100 | Simulation |
|  | 3 | Use of patient monitor and medical devices | 120 | Practice |
|  |  | Use of infusion pump | 120 | Practice |
|  | 4 | Impaired skin integrity | 100 | Lecture |
|  |  | Nursing care for patients with impaired skin integrity | 140 | Simulation |
|  | 5 | Infection control | 50 | Lecture |
|  |  | Multidrug-resistant organisms | 50 | Lecture |
|  |  | Infection control | 140 | Simulation |
|  | 6 | Transfusion nursing | 40 | Lecture |
|  |  | Transfusion nursing | 80 | Simulation |
|  |  | SBAR and handoff | 120 | Lecture & Practical |
| Advanced | 1 | Medication dosage calculation (advanced) | 40 | Lecture & Practical |
|  |  | Emergency nursing | 50 | Lecture |
|  |  | Use of defibrillator | 80 | Practice |
|  |  | Endotracheal intubation | 100 | Practice |
|  |  | Team CPR | 120 | Simulation |
|  | 2 | Oxygen therapy | 80 | Lecture |
|  |  | Non-invasive positive pressure ventilation | 80 | Lecture |
|  |  | Arterial blood gas analysis | 80 | Lecture |
|  |  | Respiratory nursing care | 160 | Simulation |
|  | 3 | Fluid therapy | 80 | Lecture |
|  |  | Acute renal failure | 100 | Lecture |
|  |  | Nursing care for dialysis patients | 80 | Lecture |
|  |  | Renal nursing care | 140 | Simulation |
|  | 4 | Normal electrocardiogram (ECG) | 60 | Lecture |
|  |  | Arrhythmia | 80 | Lecture |
|  |  | Principles and understanding of pacemaker | 50 | Lecture |
|  |  | Hemodynamic monitoring | 80 | Lecture & Practical |
|  |  | Cardiovascular nursing care | 120 | Simulation |
| *Note.* SCCDP=Stepwise clinical competency development program; SBAR=Situation, Background, Assessment, Recommendation; CPR=Cardiopulmonary resuscitation. | | | | |

[Insert Fig 2 here]

**Data Collection**

The data collection period for this study spanned from 4 September 2023 to 31 July 2024, with data collected at four points: before the program began, immediately after program completion, and at three and six months following program completion. Prior to the start of the program, permission was obtained from the nursing department of the study hospital to post recruitment posters in each department. Participants were recruited through open enrollment. Before data collection, the purpose and procedures of the study were explained to the participants, and signed consent for voluntary participation was obtained. Data collection and program sessions were conducted in the hospital’s designated training room and auditorium. A research assistant conducted a preliminary survey.

**Instruments**

To assess changes over time in participants following their involvement in the SCCDP, their general characteristics, clinical knowledge (basic and advanced), clinical performance ability, and self-efficacy were measured.

**Clinical knowledge**

The clinical knowledge assessment tool, developed by seven clinical nurse educators, was used to evaluate the knowledge of the main content covered in each session of the basic and advanced stages. This tool consisted of 30 questions at the basic level and 40 questions at the advanced level. Each item was scored 0 for an incorrect answer and 1 for a correct answer, yielding a score ranging from 0 to 30 points at the basic level and from 0 to 40 points at the advanced level. Higher scores- what was considered higher? indicate greater clinical knowledge at each stage. After development, the tool was validated by three nursing professors and three nursing managers with master’s degrees or higher, resulting in a CVI of 1.0. The tool demonstrated a 95% confidence interval (CI) of 22.72–22.90 for the basic level and 25.79–27.35 for the advanced level. The reliability of the tool, expressed as a CI value, indicates the precision of the average estimate, with narrower intervals reflecting greater accuracy [20].

**Clinical performance ability**

Clinical performance ability was assessed using the Korean Nursing Core Competency Measurement Tool (KNCCS) developed by Lee et al. [21]. This tool comprises 70 questions divided into subdomains: 12 questions on critical thinking, 21 questions on clinical performance, 13 questions on communication ability, 10 questions on understanding of humans, and 14 questions on professionalism and ethics. Responses are measured on a Likert scale ranging from 1 (“I cannot perform at all”) to 5 (“I can perform with great confidence”), with higher scores indicating greater clinical performance ability. The tool’s reliability at the time of development was Cronbach’s α = .97, and in this study, it was Cronbach’s α = .98.

**Self-efficacy**

Self-efficacy was assessed using an instrument developed by Sherer et al. [22] and later modified and supplemented by Lee [23]. This tool comprises 17 items and is measured on a Likert scale ranging from 1 (“not at all”) to 5 (“very much”). Higher scores indicate greater self-efficacy. The reliability of the tool was Cronbach’s α = .94 in Lee’s study and Cronbach’s α = .93 in this study.

**Data analysis**

The collected data were analyzed using SPSS for Windows (version 26.0; SPSS Inc., Chicago, IL, USA). The main variables were tested for normal distribution using the Kolmogorov-Smirnov test. The general characteristics of the participants were analyzed using frequencies, percentages, means, and standard deviations. Clinical knowledge, clinical performance ability, and self-efficacy before and after participation in the SCCDP were analyzed using repeated-measures ANOVA when the assumption of normality was satisfied. If the sphericity assumption was violated, the Greenhouse-Geisser correction was applied. For non-parametric data, Friedman’s ANOVA was used. Cohen’s effect sizes were calculated to determine the magnitude of the changes over time. Reliability of the measured variables was assessed using Cronbach’s α coefficient and the 95% CI of the mean.

**Ethical considerations**

This study was conducted after receiving approval (blinded for review) from the Institutional Review Board of C University Hospital, and written informed consent was obtained from all participants. The voluntary nature, anonymity, and confidentiality of participation were explained to the participants, who were informed of their right to withdraw from the study at any time without penalty. Data confidentiality and anonymity were maintained throughout the study.

**Results**

**General characteristics of the participants**

The general characteristics of the participants are shown in Table 2. A total of 49 new nurses participated in this study, with an average age of 23.65 ± 1.95 years; 38 participants (77.6%) were under 24 years old, and 11 participants (22.4%) were between 24 and 30 years old. The majority of the participants were women (n = 37, 75.5%). Most participants were single (n = 48, 98.0%), with only one (2.0%) married. By department, the emergency and operating rooms had the highest representation with 18 participants (36.7%), followed by the internal medicine ward (n=17, 34.7%), the surgical ward (n = 6, 12.2%), the surgical intensive care unit (n = 5, 10.2%), and the internal medicine intensive care unit (n = 3, 6.1%).

| **Table 2. The general characteristics of the participants** (n=49) | | |
| --- | --- | --- |
| **Characteristics** | **Categories** | **n (%) or M±SD** |
| Age (years) | ≤24 | 38 (77.6) |
|  | >24~≤30 | 11 (22.4) |
|  | Total | 23.65±1.95 |
| Gender | Male | 12 (24.5) |
|  | Female | 37 (75.5) |
| Marriage | Single | 48 (98.0) |
|  | Married | 1 (2.0) |
| Department | Medical ward | 17 (34.7) |
|  | Surgical ward | 6 (12.2) |
|  | Medical ICU | 3 (6.1) |
|  | Surgical ICU | 5 (10.2) |
|  | ER, OR | 18 (36.7) |
| *Note.* M=Mean; SD=Standard Deviation; ICU=Intensive Care Unit; ER=Emergency Room; OR=Operation Room. | | |

**Effectiveness of the SCCDP for new nurses**

The effectiveness of the SCCDP for new nurses is presented in Table 3. Basic clinical knowledge improved significantly immediately and three and six months after program participation, compared to pre-program levels (F = 40.01, *p* < .001). Among the subareas, significant improvements in medical device operation (F = 72.63, *p* < .001), infection control, multidrug-resistant organisms (F = 26.28, *p* < .001), and skin care (F = 13.39, *p* = .004) were observed. Notably, the area of medical device operation demonstrated the largest effect size (ES = 1.56 – 1.65). Conversely, no significant changes were observed in the SBAR and handover areas (F = 3.80, *p* = .284). Advanced clinical knowledge also showed significant improvement immediately and at three and six months after program participation, compared to pre-program levels (F = 26.06, *p* < .001). The greatest improvement was observed immediately after the program (ES = 1.84) and remained at a significantly high level three and six months after. Among the subdomains, emergency nursing (F = 33.42, *p* < .001), respiratory system nursing (F = 29.61, *p* < .001), renal system nursing (F = 34.63, *p* < .001), and circulatory system nursing (F = 8.56, *p* < .001), all showed significant improvements. The analysis for “Circulatory system nursing” includes 47 participants, excluding 2 who missed the lecture and related tasks. Emergency nursing particularly demonstrated the largest effect size immediately after the program (ES = 1.22). Clinical performance ability improved significantly three and six months following program participation compared to pre-program levels (χ² = 55.92, *p* < .001). Significant improvements were observed across all sub-domains, including interpersonal relationships and communication (χ² = 107.59, *p* < .001), professional attitude (χ² = 77.57, *p* < .001), critical thinking and evaluation (χ² = 43.67, *p* < .001), general clinical performance (χ² = 35.51, *p* < .001), and specialized clinical performance (χ² = 11.50, *p* < .001). In particular, the interpersonal and communication demonstrated the largest effect size (ES = 2.05–2.09). Self-efficacy showed a slight increase at both three and six months following program participation compared to pre-program levels; however, the change was not statistically significant (F = 2.80, *p* = .066).

From the tools suggested in the methods, the table below doesn’t satidfactority represent the results. For example clinical competence had 30 and 40 items- the table shows few- were they later clustered? It is not clear

| Table 3. Changes in the effectiveness of stepwise clinical competency development program for new nurses over time (n = 49) | | | | | | | | | | |
| --- | --- | --- | --- | --- | --- | --- | --- | --- | --- | --- |
| Variables | Pre^a^ | Post 1^b^ | Post 2^c^ | Post 3^d^ | Source | F/χ^2^ (*p*) | Effect size^a-b^  (CI) | Effect size^a-c^  (CI) | Effect size^a-d^  (CI) |  |
|  | Mean±SD | Mean±SD | Mean±SD | Mean±SD |  |  |  |  |  |  |
| Basic clinical knowledge | 22.31±2.05 | 24.65±2.06 | 24.80±2.27 | 24.53±2.22 | Time | 40.01 (<.001)^§^  Post hoc: a<b,c,d | 1.14  (0.71-1.58) | 1.15  (0.72-1.59) | 1.04  (0.61-1.47) |  |
| Intravenous injection | 2.33±0.63 | 2.27±0.73 | 2.53±0.62 | 2.51±0.58 | Time | 9.69 (.021)^§^ | -0.01  (-0.49-0.32) | 0.32  (-0.09-0.73) | 0.30  (-0.10-0.70) |  |
| Intradermal injection | 2.57±0.54 | 2.37±0.60 | 2.71±0.46 | 2.69±0.47 | Time | 14.91 (.002)^§^  Post hoc: b<c,d | -0.34  (-0.75-0.07) | 0.27  (-0.14-0.68) | 0.23  (-0.18-0.64) |  |
| Operation of medical devices | 3.02±0.66 | 4.12±0.75 | 4.23±0.80 | 4.06±0.78 | Time | 72.63 (<.001)^§^  Post hoc: a<b,c,d | 1.56  (1.10-2.02) | 1.65  (1.18-2.12) | 1.44  (0.99-1.89) |  |
| Skin care | 3.65±0.97 | 4.23±0.84 | 3.74±1.10 | 3.71±1.04 | Time | 13.39 (.004)^§^  Post hoc: a<b, b>d | 0.64  (0.23-1.05) | 0.09  (-0.32-0.49) | 0.06  (-0.35-0.46) |  |
| Infection control & multidrug-resistant organisms | 2.98±0.99 | 3.61±0.81 | 3.88±0.83 | 3.78±0.92 | Time | 26.28 (<.001)^§^  Post hoc: a<b,c,d | 0.70  (0.28-1.11) | 0.99  (0.56-1.41) | 0.84  (0.42-1.26) |  |
| Blood transfusion nursing | 4.63±0.53 | 4.76±0.43 | 4.43±0.71 | 4.53±0.54 | Time | 11.59 (.009)^§^  Post hoc: b>c | 0.27  (-0.14-0.68) | -0.32  (-0.73-0.09) | -0.19  (-0.59-0.22) |  |
| SBAR & handover | 3.12±0.53 | 3.27±0.45 | 3.29±0.50 | 3.25±0.52 | Time | 3.80 (.284)^§^ | 0.31  (-0.10-0.71) | 0.33  (-0.08-0.74) | 0.25  (-0.16-0.65) |  |
| Advanced clinical knowledge | 26.57±2.72 | 31.86±3.01 | 29.12±3.78 | 28.53±2.84 | Time | 26.06 (<.001)  Post hoc: a<b,c,d, b>c,d | 1.84  (1.36-2.33) | 0.77  (0.36-1.19) | 0.71  (0.29-1.12) |  |
| Emergency system nursing | 6.67±1.31 | 8.18±1.17 | 7.00±1.31 | 6.71±1.41 | Time | 33.42 (<.001)^§^  Post hoc: a<b, b>c,d | 1.22  (0.78-1.66) | 0.25  (-0.15-0.66) | 0.03  (-0.38-0.43) |  |
| Respiratory system nursing | 6.29±1.44 | 7.74±1.10 | 7.08±1.24 | 7.00±1.10 | Time | 29.61 (<.001)^§^  Post hoc: a<b, b>c,d | 1.13  (0.70-1.57) | 0.59  (0.18-1.00) | 0.55  (0.14-0.97) |  |
| Renal system nursing | 6.65±1.11 | 8.14±1.06 | 7.31±1.50 | 6.92±1.30 | Time | 34.63 (<.001)^§^  Post hoc: a<b, b>c,d | 1.37  (0.92-1.82) | 0.50  (0.09-0.91) | 0.22  (-0.18-0.63) |  |
| Circulatory system nursing^*^ | 6.94±1.74 | 8.13±1.39 | 7.68±1.42 | 7.85±1.18 | Time | 8.56 (<.001)^￡^  Post hoc: a<b,d | 0.76  (0.34-1.17) | 0.47  (0.06-0.88) | 0.61  (0.20-1.03) |  |
| Clinical performance ability | 2.94±0.57 | - | 3.82±0.46 | 3.82±0.48 | Time | 55.92 (<.001)^§^  Post hoc: a<c,d | - | 1.70  (1.23-2.17) | 1.67  (1.20-2.14) |  |
| Interpersonal relationships and communication | 2.67±0.63 | - | 3.81±0.47 | 3.84±0.48 | Time | 107.59 (<.001)^￡^  Post hoc: a<c,d | - | 2.05  (1.55-2.55) | 2.09  (1.59-2.59) |  |
| Professional attitude | 2.57±0.66 | - | 3.63±0.58 | 3.69±0.53 | Time | 77.57 (<.001)^￡^  Post hoc: a<c,d |  | 1.71  (1.23-2.18) | 1.87  (1.39-2.36) |  |
| Critical thinking and evaluation | 3.22±0.65 | - | 3.93±0.46 | 3.88±0.50 | Time | 43.67 (<.001)^§^  Post hoc: a<c,d |  | 1.26  (0.82-1.70) | 1.34  (0.70-1.57) |  |
| General clinical performance | 3.17±0.69 | - | 3.90±0.50 | 3.87±0.53 | Time | 35.51 (<.001)^§^  Post hoc: a<c,d |  | 1.21  (0.77-1.65) | 1.14  (0.70-1.57) |  |
| Specialized clinical performance | 3.34±0.68 | - | 3.81±0.61 | 3.82±0.66 | Time | 11.50 (<.001)^￡^  Post hoc: a<c,d |  | 0.73  (0.31-1.15) | 0.72  (0.30-1.13) |  |
| Self-efficacy | 65.47±8.97 | - | 68.57±9.94 | 68.04±10.40 | Time | 2.80 (.066) |  | 0.33  (-0.08-0.73) | 0.27  (-0.14-0.67) |  |
| *Note.* Pre=Pre-program; Post 1=Immediately after program; Post 2=3 months after program; Post 3=6 months after program; SD=Standard deviation; SBAR=Situation, Background, Assessment, Recommendation; §Friedman’s ANOVA; ￡Greenhouse-Geisser; ^*^Although 49 participants were registered in the study, only 47 were included in the analysis for “Circulatory system nursing” because 2 participants did not attend the circulatory system lecture due to scheduling conflicts. | | | | | | | | | | |

**Discussion**

This study developed and implemented an SCCDP to improve the clinical performance of new nurses, support their effective adaptation to clinical settings, and increase their intention to remain in the workplace. To assess its effectiveness, the SCCDP was structured into basic and advanced stages and applied over time. Clinical knowledge, clinical performance ability, and self-efficacy were evaluated to confirm the program’s effectiveness. Verification of the effectiveness of the SCCDP revealed improvements in both basic and advanced clinical knowledge and clinical performance ability following training. Notably, the SCCDP demonstrated a lasting impact, with effects sustained immediately after implementation and continuing at three and six months post-training. This is more thoroughly discussed below.

The SCCDP is a systematic educational program that combines theoretical lectures, practical training, and simulations, effectively contributing to the strengthening of practice-oriented competencies among new nurses. Education led by clinical nurse educators has been positively evaluated before, as it enables systematic and level-specific instructions, which has been proven to reduce anxiety among new nurses [6, 24]. The amateur nursing skills of new nurses, challenges stemming from the gap between theoretical content and practice, and pressure to perform well contribute to psychological anxiety [25]. Furthermore, improper education that does not teach the appropriate skill levels may widen this gap. To address this, retention rates may be improved by providing training that combines practice and simulations tailored to the developmental and adaptation stages of new nurses [11]. In addition, the gap between the theoretical knowledge required at each stage and practical applications in clinical settings can be minimized, and simulation practice based on real cases can enhance learning confidence while reinforcing critical thinking skills [26]. Step-by-step education is essential for new nurses who engage in intensive work over time [13, 24]. This program was designed to help new nurses strengthen their clinical knowledge and enhance their ability to apply it through repetitive learning and practice over a defined period. To provide standardized education for new nurses, it is necessary to measure the program’s effectiveness by expanding its application across medical institutions.

The results of this study revealed that the basic clinical knowledge of new nurses significantly improved immediately and three and six months after the program. Their ability to operate medical devices improved the most. These suggest that the new nurses learned the correct usage of various medical devices they handle in the field, enhancing their ability to operate them safely. Of the participants, 53.0% worked primarily in emergency rooms, operating rooms, and intensive care units, where they frequently used various high-risk medical devices. Through repeated use, they are likely to become more proficient in operating these devices over time, thereby contributing to improved clinical knowledge. New nurses with one to three months of experience were found to frequently make mistakes and face difficulties when using unfamiliar medical devices [13], with a high incidence of safety accidents related to these devices [27]. Since the use of inappropriate equipment can result in patient safety incidents, it is essential to provide practical training on equipment use at the start of employment. As this is a critical competency for enhancing patient safety and improving nurses’ work efficiency, it should become an integral component of the initial clinical adaptation process for new nurses. It is also crucial to develop a simulation program based on error events that occur in real clinical settings.

Advanced clinical knowledge was also sustained over time, with the greatest impact in emergency nursing. This indicates that the new nurses effectively acquired the specialized skills and knowledge required for emergency situations, significantly enhancing their ability to respond to emergencies in real clinical settings. Since nurses serve as the first responders in emergency situations, they require complex competencies, including specialized knowledge, quick judgment, and strong decision-making skills, to respond effectively [28, 29]. Emergency nursing is one of the most challenging areas frequently encountered by new nurses. A lack of experience in handling emergency situations can reduce confidence in patient care. Therefore, it is crucial to provide education that incorporates simulation-based training [28]. Experience in responding to emergency situations provides a sense of security and confidence as it helps individuals understand the complexities of treatments and procedures in emergency contexts [30]. The findings reveal that the educational program enhanced new nurses’ understanding of emergency nursing, developed their ability to prevent the worsening of patients’ conditions, and delivered effective emergency care through simulation exercises.

The results of this study demonstrate that the greatest effect among clinical performance abilities was observed in interpersonal relationships and communication. This indicates that the program enhanced nurses’ ability to collaborate effectively within the team through clear communication with patients, families, and other medical staff. Nurses spend the most time with patients, making interpersonal and communication skills essential competencies. Therefore, education aimed at improving these skills is necessary [31, 32]. Communication competency is considered an essential skill for nurses to provide safe patient-centered care in clinical settings. However, no significant changes were observed in the SBAR and handover capabilities. Handover is a critical process for exchanging information between nurses and an important factor that can impact patient care. SBAR-based handovers were found to enhance empathy and trust between nurses and patients and improve responsiveness to questions and requests from patients or their families, thereby strengthening communication among medical staff as well [33]. However, this difference in findings may be because the time allocated to intensive education in this area was relatively insufficient. New nurses find handovers challenging and often feel frustrated because of their limited ability to conduct them efficiently [34, 35]. To maximize knowledge and skills and enhance competency and confidence, repeated education, simulation training, and a continuous feedback system should be provided.

Self-efficacy showed a slight increase following the program; however, this change was not statistically significant. This finding suggests that self-efficacy may be a capacity for new nurses to develop independently; they may gain confidence through psychological support, feedback, and accumulation of clinical experience, separate from the acquisition of clinical skills and knowledge. Additionally, self-efficacy is associated with an individual’s positive response to challenges [36], which may explain why the increasing workload and high role expectations experienced by new nurses contribute to an increased sense of burden over time [37]. For new nurses, the transition period from student to nurse is critical for professional growth and development. To enhance self-efficacy through positive experiences and appropriate support, continuous mentoring, accumulation of successful experiences, and psychological support are needed alongside educational programs [13, 38].

This study proposes an effective program to strengthen the clinical competency and practice adaptation of new nurses. However, it has several limitations. First, additional educational interventions are required to further develop SBAR and handover skills. Second, educational strategies that incorporate long-term mentoring and continuous feedback are necessary to enhance psychological competencies such as self-efficacy. Third, since our study used a convenient sample from a single medical institution, caution must be exercised when generalizing the findings. Additionally, as all three instruments—clinical knowledge, clinical performance ability, and self-efficacy—were administered simultaneously at each measurement point, participants may have experienced fatigue due to the length of the questionnaires. This potential burden may have influenced the accuracy or completeness of responses, and should be considered a limitation of the study. Additionally, the absence of a control group limits the ability to determine causality in observed improvements. Future studies should consider employing controlled or randomized trial designs to more rigorously evaluate the program's effectiveness. In the future, the program should be expanded and applied across diverse hospitals and clinical settings and continuously improved through feedback.

**Conclusion**

The SCCDP developed in this study combines theoretical content, practical training, and simulations. This effectively improved the basic and advanced clinical knowledge and clinical performance abilities of new nurses, and these improvements were sustained over time. This program provides an opportunity to bridge the gap between theoretical knowledge and practical applications, enabling nurses to effectively apply the learned concepts in real clinical settings. In the long term, the SCCDP helped new nurses to better adapt to the clinical environment. The program developed in this study can be utilized to enhance the clinical adaptation and competency of new nurses, potentially contributing to reducing nurse turnover and providing safer and more effective nursing care in clinical settings. How sustainable is the programme?

Does it also mean the nurses at employment aren’t qualified for the profession they were trained for?

**Acknowledgments**

The authors express their heartfelt appreciation to the participants and thank them for their valuable time and support.

**References**

1. Hospital Nurses Association. A survey on the current status of hospital nursing staff. 2022. Available from: https://khna.or.kr/home/pds/utilities.php?bo_table=board1&wr_id=8115 [Accessed 15 June 2023].

2. Ulupinar S, Aydogan Y. New graduate nurses' satisfaction, adaptation and intention to leave in their first year: a descriptive study. J Nurs Manag. 2021;29(6):1830-1840. doi: 10.1111/jonm.13296

3. NSI Nursing Solutions Inc. 2021 NSI national health care retention & RN staffing report. 2021. Available from: https://www.nsinursingsolutions.com/Documents/Library/NSI_National_Health_Care_Retention_Report.pdf [Accessed 24 February 2023].

4. Kim SH, Kim JS. Predictors of emotional labor, interpersonal relationship, turnover intention and self-efficacy on job stress in new nurses. J Digit Converg. 2020;18(11):547-558. doi: 10.14400/JDC.2020.18.11.547

5. Kim MJ, Shin SJ, Lee IY. Education programs for newly graduated nurses in hospitals: a scoping review. Korean J Adult Nurs. 2020;32(5):440-454. doi: 10.7475/kjan.2020.32.5.440

6. Coventry TH, Russell KP. Clinical sympathy – a mixed method study of the relationship between the clinical nurse educator and the graduate nurse. Nurse Educ Pract. 2021;55:103150. doi: 10.1016/j.nepr.2021.103150

7. Kwon IG, Cho YA, Kim KS, Kim MS, Cho MS. Development of Korean nurse residency program for tertiary hospitals. J Korean Clin Nurs Res. 2021;27(2):149-164. doi: 10.22650/JKCNR.2021.27.2.149

8. Jeong HW, Moon SH, Ju D, Seon SH, Kang N. Effects of the clinical core competency empowerment program for new graduate nurses led by clinical nurse educator. Crisisonomy. 2021;17(6):109-123. doi: 10.14251/crisisonomy.2021.17.6.109

9. Shin SJ, Park YW, Kim MJ, Kim JH, Lee IY. Survey on the education system for new graduate nurses in hospitals: focusing on the preceptorship. Korean Med Educ Rev. 2019;21(2):112-122. doi: 10.17496/kmer.2019.21.2.112

10. Kim YK. Need analysis of standardized education and training program development to practical competency reinforcement programs for new nurses. J Humanit Soc Sci. 2021;12(5):1727-1740. doi: 10.22143/HSS21.12.5.122

11. Choi H, Shin S. Quality of clinical nursing education for new graduate nurses: A concept analysis with a hybrid model. J Korean Acad Soc Nurs Educ. 2023;29(1):27-40. doi: 10.5977/jkasne.2023.29.1.27

12. Kwon SH, Kim SH, Jung SY, Mo MH, Chun YM, Hong SY, et al. Core nursing skills and suggestions for improvement in clinical teaching for new graduate nurses as recognized by clinical nurse educators. J Korean Data Inf Sci Soc. 2021;32(3):309-324. doi: 10.7465/jkdi.2021.32.2.309

13. Ahn SH, Jeong HW, Yang SG, Jung US, Choi ML, Kim HS. Temporal exploration of new nurses’ field adaptation using text network analysis. J Korean Acad Nurs. 2024;54(3):358-371. doi: 10.4040/jkan.24034

14. Chen S, Zhang C, Li W. The effects of competency-based training model in the training of new nurses: a meta-analysis and systematic review. PLoS One. 2022;17(11):e0277484. doi: 10.1371/journal.pone.0277484

15. Lewis LS, Rebeschi LM, Hunt E. Nursing education practice update 2022: competency-based education in nursing. SAGE Open Nurs. 2022;8. doi: 10.1177/23779608221140774

16. Nagai M, Oikawa M, Komagata T, Basuana JDB, Ulyabo GK, Minagawa Y, et al. Clinical competency of nurses trained in competency-based versus objective-based education in the Democratic Republic of the Congo: a qualitative study. Hum Resour Health. 2024;22:38. doi: 10.1186/s12960-024-00921-0

17. Frank JR, Mungroo R, Ahmad Y, Wang M, De Rossi S, Horsley T. Toward a definition of competency-based education in medicine: a systematic review of published definitions. Med Teach. 2010;32(8):631-637. doi: 10.3109/0142159X.2010.500898

18. Kim J, Park JH, Shin S. Effectiveness of simulation-based nursing education depending on fidelity: a meta-analysis. BMC Med Educ. 2016;16(1):152. doi: 10.1186/s12909-016-0672-7

19. James AH. Valuing the emotions of leadership learning experience in nursing education. Nurse Educ Pract. 2023;71, 103716. doi: 10.1016/j.nepr.2023.103716

20. El-Uri FI, Malas N. Analysis of use of a single best answer format in an undergraduate medical examination. Qatar Med J. 2013;2013(1):1. doi: 10.5339/qmj.2013.1

21. Lee WH, Kim S, An J. Development and evaluation of Korean nurses’ core competency scale (KNCCS). Open J Nurs. 2017;7(5):599-613. doi: 10.4236/ojn.2017.75045

22. Sherer M, Maddux JE, Mercandante B, Prentice-Dunn S, Jacobs B, Rogers RW. The self-efficacy scale: construction and validation. Psychol Rep. 1982;51(2):663-671.

23. Lee JH. A study on the predictors of health promotion behavior among nurses working at medical centers [Master’s thesis]. Seoul: Korea University; 1998.

24. Kim S, Shin S, Lee I. Exploring the roles and outcomes of nurse educators in hospitals: a scoping review. Korean Med Educ Rev. 2023;25(1):55-67. doi: 10.17496/kmer.22.026

25. Hwang H, Lee Y. Newly graduated nurse’s resilience experience. J Korea Contents Assoc. 2021;21(10):656-667. doi: 10.5392/JKCA.2021.21.10.656

26. Seo JY, Choi EH. Effects of simulation practice according to critical thinking disposition in nursing students. J Korean Data Inf Sci Soc. 2020;31(3):559-568. doi: 10.7465/jkdi.2020.31.3.559

27. Hur HK, Kim KK, Jung JS. A systematic content analysis of the closed nursing malpractice claims related to patients’ safety. J Korean Acad Nurs Adm. 2023;29(2):99-108. doi: 10.11111/jkana.2023.29.2.99

28. Kim J, Lee M, Lee M. Implementation and evaluation of an emergency response training program for newly graduated nurses: a scoping review. J Korean Soc Simul Nurs. 2024;12(1):59-72. doi: 10.17333/JKSSN.2024.12.1.59

29. Miranda FBG, Alves G, Mazzo A. Competences in the training of nurses to assist the airway of adult patients in urgency and emergency situations. Rev Lat-Am Enferm. 2021;29:e3434. doi: 10.1590/1518-8345.3380.3434

30. Towner EC, East LS, Lea J. The experiences of new graduate nurses caring for the deteriorating patient in rural areas: an integrative review. Collegian. 2022;29(2):245-251. doi: 10.1016/j.colegn.2021.12.006

31. Godsey JA, Houghton DM, Hayes T. Registered nurse perceptions of factors contributing to the inconsistent brand image of the nursing profession. Nurs Outlook. 2020;68(6):808-821. doi: 10.1016/j.outlook.2020.06.005

32. Kerr D, Ostaszkiewicz J, Dunning T, Martin P. The effectiveness of training interventions on nurses' communication skills: a systematic review. Nurse Educ Today. 2020;89:104405. doi: 10.1016/j.nedt.2020.104405

33. Abbaszade A, Assarroudi A, Armat MR, Stewart JJ, Rakhshani MH, Sefidi N, et al. Evaluation of the impact of hand off based on the SBAR technique on quality of nursing care. J Nurs Care Qual. 2021;36(3):E38-E43. doi: 10.1097/ncq.0000000000000498

34. Chung JYS, Li WHC, Ho LLK, Cheung AT, Chung JOK. Newly graduate nurse perception and experience of clinical handover. Nurse Educ Today. 2021;97:104693. doi: 10.1016/j.nedt.2020.104693

35. Park S, Lee JL. Research trend analysis of Korean new graduate nurses using topic modeling. J Korean Acad Soc Nurs Educ. 2021;27(3):240-250. doi: 10.5977/jkasne.2021.27.3.240

36. Peñacoba C, Catala P, Velasco L, Carmona-Monge FJ, Garcia-Hedrera FJ, Gil-Almagro F. Stress and quality of life of intensive care nurses during the COVID-19 pandemic: self-efficacy and resilience as resources. Nurs Critical Care. 2021;26(6):493-500. doi: 10.1111/nicc.12690

37. Kim JH, Shin HS. Exploring barriers and facilitators for successful transition in new graduate nurses: a mixed methods study. J Prof Nurs. 2020;36(6):560-568. doi: 10.1016/j.profnurs.2020.08.006

38. Jangland E, Gunningberg L, Nyholm L. A mentoring programme to meet newly graduated nurses' needs and give senior nurses a new career opportunity: a multiple-case study. Nurse Educ Pract. 2021;57:103233. doi: 10.1016/j.nepr.2021.103233
