# Supplementary material for: Development and evaluation of a stepwise clinical competency development program for new nurses: A single-group repeated-measures quasi-experimental study
Source: PLoS One. 2026 Feb 9;21(2):e0342464. doi: 10.1371/journal.pone.0342464 (PMC12885250; doi:10.1371/journal.pone.0342464)
Supplement: Attachment — Submitted filename: Response to Reviewer #1.docx [file pone.0342464.s004.docx]

**Dear Reviewer,**

Thank you for your insightful feedback on our manuscript. We greatly value your thorough review and have made the necessary revisions in response to your comments. We hope that these adjustments meet your expectations.

| **Comment** | **Revision** | |
| --- | --- | --- |
|  | **Before** | **After** |
| The necessity of conducting this research is not clearly defined. Please provide a clearer rationale for your study. | New nurses experience challenges in adapting to the hospital’s organizational environment, often due to insufficient knowledge and professional skills, excessive workload, and difficulties in communication and collaboration with other medical staff and colleagues [2]. Nurse turnover leads to significant financial losses for hospitals [3], with new nurse turnover comprising the largest portion of the overall nursing attrition [1], which further increases the burden on patients and remaining staff. To support new nurses in adapting effectively to the clinical setting, organizations must implement strategies that reduce turnover intention and job stress while enhancing self-efficacy [4]. | Thank you for this valuable comment. As suggested, we clarified the rationale and necessity of this research more explicitly in the Introduction section.:  Although previous research highlights the need to support new nurses' clinical adaptation through education and structured training [4, 5], existing training programs are often inconsistent, insufficiently standardized, and lack long-term evaluations of effectiveness [6, 7]. Thus, developing a structured, evidence-based educational intervention is crucial to systematically address the competency gaps and adaptation challenges faced by new nurses, ultimately aiming to reduce turnover and improve patient care outcomes [8, 9]. |
| In the methods section, clarify why the study was conducted as a quasi-experimental study. | This study employed a single-group, repeated-measures quasi-experimental design to assess the impact of the SCCDP on new nurses’ basic and advanced practical knowledge, clinical performance ability, and self-efficacy. | As you pointed out, we have clarified the rationale for selecting the research design. Thank you for your valuable suggestion.:  This study employed a single-group, repeated-measures quasi-experimental design to assess the impact of the SCCDP on new nurses’ basic and advanced practical knowledge, clinical performance ability, and self-efficacy. This research was conducted at a single hospital, aiming to provide structured, systematic education for newly recruited nurses joining the institution. Due to ethical considerations and the necessity of providing standardized training to all new nurses at designated intervals, it was not feasible to include a control group in this study. |
| At the end of the discussion, please include suggestions for future research. | In the future, the program should be expanded and applied across diverse hospitals and clinical settings and continuously improved through feedback. | As suggested, we added clear suggestions for future research at the end of the discussion section. Thank you for your valuable comment.:  In the future, the program should be expanded and applied across diverse hospitals and clinical settings and continuously improved through feedback. Additionally, future research should utilize controlled or randomized trial designs to rigorously evaluate the causality and generalizability of the program's effectiveness. Longitudinal research with multi-interval follow-ups would be particularly beneficial to comprehensively understand the long-term impact of the program on clinical competency and adaptation outcomes. Further qualitative studies exploring participants' experiences may also provide deeper insights into factors affecting clinical competency development and adaptation processes among new nurses. |
| After the conclusion, declare any conflicts of interest related to the study. | None | As requested, we have clearly declared any conflicts of interest after the conclusion. Thank you for this suggestion.:  **Conflicts of Interest**  The authors declare that they have no conflicts of interest associated with this study |
