# Supplementary material for: Development and evaluation of a stepwise clinical competency development program for new nurses: A single-group repeated-measures quasi-experimental study
Source: PLoS One. 2026 Feb 9;21(2):e0342464. doi: 10.1371/journal.pone.0342464 (PMC12885250; doi:10.1371/journal.pone.0342464)
Supplement: Attachment — Submitted filename: Response to Reviewer #4_v4.docx [file pone.0342464.s005.docx]

**Dear Reviewer,**

Thank you for your insightful feedback on our manuscript. We greatly value your thorough review and have made the necessary revisions in response to your comments. We hope that these adjustments meet your expectations.

| **Comment** | **Revision** | |
| --- | --- | --- |
|  | **Before** | **After** |
| Consider a remaining the conceptual framework a theoretical framework - you are emebding the study on CBE model | **Conceptual framework** The SCCDP for new nurses was structured based on the theoretical content of competency-based education (CBE) (Fig 1). CBE is an educational approach focused on ensuring that learners acquire specific competencies and effectively apply them to real-life situations [20]. This conceptual framework is designed to enhance the clinical adaptation and competency of new nurses and incorporates several key elements. | We thank the reviewer for this helpful comment. :  **Theoretical framework**  The SCCDP for new nurses was structured based on the foundations of competency-based education (CBE) (Fig 1). CBE is an educational approach focused on ensuring that learners acquire specific competencies and effectively apply them to real-life situations [20]. This theoretical framework is designed to enhance the clinical adaptation and competency of new nurses and incorporates several key elements. |
| Does the study have any recommendation to training institutitions? or clinical learning among students within your hospital? - considering you had to train for soo many hours after graduating from college- Are there deficits from Curricula? Is clinical teaching and learning for nursing students deficient? | None | We thank the reviewer for raising this important point regarding the broader educational implications of our findings. In the revised Discussion, we added a section emphasizing recommendations for training institutions and nursing curricula.:  In addition, the findings carry broader implications for nursing education and workforce policy. In Korea, undergraduate nursing curricula have traditionally emphasized theoretical instruction, which may not sufficiently prepare students for the complexities of clinical practice. Our results highlight the need for curricula to incorporate authentic clinical scenarios and practice-oriented processes, developed in close collaboration with hospital-based practitioners and academic nurse educators. Establishing regular joint consultations between these groups could help align competencies, standardize expectations, and promote scenario-based learning. Furthermore, the prolonged employment waiting periods faced by many new graduates—sometimes lasting one to two years—can create knowledge attrition and skill gaps. This not only underscores the necessity of structured bridging or refresher programs but also calls for policy-level discussions to improve workforce planning and facilitate smoother transitions from education to practice. It is also noteworthy that within undergraduate curricula, theoretical courses are often allocated relatively more weight compared with practice-based courses such as simulation. Although this reflects institutional credit policies rather than individual teaching choices, such structures may inadvertently devalue practice-oriented education and discourage greater emphasis on experiential learning. Addressing these imbalances through curricular reform and stronger university–hospital collaboration could enhance the effectiveness of simulation and practice-based training in preparing graduates for the realities of clinical practice. |
| Separate the sustainability and generazability of the findings from conclusion- Have it as a subheading below it Limitations of the study can be included and allude to the generazability and scalability of the findings | **Conclusion**  The SCCDP developed in this study combines theoretical content, practical training, and simulations. This effectively improved the basic and advanced clinical knowledge and clinical performance abilities of new nurses, and these improvements were sustained over time. This program provides an opportunity to bridge the gap between theoretical knowledge and practical applications, enabling nurses to effectively apply the learned concepts in real clinical settings. In the long term, the SCCDP helped new nurses to better adapt to the clinical environment. Regarding sustainability, the SCCDP was systematically integrated into the existing hospital education structure, utilizing internal resources such as clinical nurse educators and standardized training protocols, making the program feasible and sustainable for long-term implementation. Additionally, this study highlights the necessity of structured support programs that help newly graduated nurses effectively integrate and apply their theoretical knowledge within complex clinical settings, thereby facilitating a smoother transition from academic learning to professional practice. The program developed in this study can be utilized to enhance the clinical adaptation and competency of new nurses, potentially contributing to reducing nurse turnover and providing safer and more effective nursing care in clinical settings. | We thank the reviewer for the constructive suggestion.:  **Limitations and Implications**  This study has several limitations. First, it was conducted in a single medical institution using a convenience sample, which restricts the generalizability of the findings. Second, the absence of a control group prevents clear causal inference. Third, the simultaneous administration of lengthy instruments may have induced survey fatigue, potentially influencing the accuracy of responses.  Despite these limitations, the SCCDP demonstrates important implications for sustainability and scalability. The program was systematically integrated into the hospital’s existing educational infrastructure, utilizing clinical nurse educators and standardized training protocols, which suggests its feasibility for long-term implementation. To enhance generalizability, the SCCDP should be evaluated in diverse hospital settings and across different regions, ideally through multicenter or randomized controlled designs.  These findings also highlight broader educational and policy implications. In Korea, undergraduate nursing curricula have traditionally emphasized theoretical courses, while practice-based courses such as simulation are often assigned fewer credits, which may inadvertently undervalue experiential learning. Although this reflects institutional credit allocation policies rather than individual teaching choices, such structures may discourage emphasis on practice-oriented education. Addressing these imbalances through curricular reforms and closer collaboration between academic institutions and hospitals could enhance the effectiveness of simulation and clinical training. In addition, the prolonged waiting periods many graduates face before entering the workforce—sometimes up to one or two years—can create knowledge attrition and skill gaps, underscoring the need for structured bridging or refresher programs and policy-level workforce planning.  Future research should pursue three directions. First, controlled or randomized trial designs are needed to rigorously evaluate causal effects of the SCCDP. Second, multicenter and cross-national studies would help assess the scalability and generalizability of the program. Third, qualitative or mixed-methods studies could provide deeper insights into new nurses’ adaptation processes and the organizational contexts influencing program effectiveness.  **Conclusion**  The SCCDP developed in this study combined theoretical content, practical training, and simulations, effectively improving both basic and advanced clinical knowledge as well as clinical performance ability among new nurses. These findings highlight the value of structured, competency-based programs in bridging the gap between academic preparation and practice, while supporting the clinical adaptation of new nurses. |
