# Supplementary material for: Development and evaluation of a stepwise clinical competency development program for new nurses: A single-group repeated-measures quasi-experimental study
Source: PLoS One. 2026 Feb 9;21(2):e0342464. doi: 10.1371/journal.pone.0342464 (PMC12885250; doi:10.1371/journal.pone.0342464)
Supplement: Attachment — Submitted filename: Response to editor.docx [file pone.0342464.s006.docx]

**Dear Reviewer,**

We sincerely appreciate your and the reviewers’ thoughtful and constructive feedback on our manuscript titled “Development and evaluation of a stepwise clinical competency development program for new nurses.”

| **Comment** | **Revision** | |
| --- | --- | --- |
|  | **Before** | **After** |
| Enhance the methodology section on the abstract | This study employed a single-group repeated-measures quasi-experimental design. From September 2023 to July 2024, 49 newly recruited nurses from C University Hospital in South Korea, participated in the SCCDP. The program was divided into basic and advanced stages and incorporated theoretical content, practice, and simulation. The outcomes measured included basic and advanced clinical knowledge, clinical performance ability, and self-efficacy at four intervals: pre-intervention, immediately post-intervention, and at three and six months post intervention. | This study employed a single-group repeated-measures quasi-experimental design. From September 2023 to July 2024, 49 new nurses from C University Hospital in South Korea participated in the SCCDP, which consisted of lectures, practice, and simulation validated by experts. The outcomes measured included basic and advanced clinical knowledge, clinical performance ability, and self-efficacy at four intervals: pre-intervention, immediately post-intervention, and at three and six months post intervention. Data were analyzed using repeated-measures and Friedman’s ANOVA, and effect sizes were calculated. |
