# Supplementary material for: Development and evaluation of a stepwise clinical competency development program for new nurses: A single-group repeated-measures quasi-experimental study
Source: PLoS One. 2026 Feb 9;21(2):e0342464. doi: 10.1371/journal.pone.0342464 (PMC12885250; doi:10.1371/journal.pone.0342464)
Supplement: Attachment — Submitted filename: Response to Reviewer #5.docx [file pone.0342464.s007.docx]

**Dear Reviewer,**

Thank you for your insightful feedback on our manuscript. We greatly value your thorough review and have made the necessary revisions in response to your comments. We hope that these adjustments meet your expectations.

| **Comment** | **Revision** | |
| --- | --- | --- |
|  | **Before** | **After** |
| Enhancing the Data Analysis section by explicitly reporting normality test outcomes and clearly stating which variables required non-parametric analysis. | The collected data were analyzed using SPSS for Windows (version 26.0; SPSS Inc., Chicago, IL, USA). The main variables were tested for normal distribution using the Kolmogorov-Smirnov test. The general characteristics of the participants were analyzed using frequencies, percentages, means, and standard deviations. Clinical knowledge, clinical performance ability, and self-efficacy before and after participation in the SCCDP were analyzed using repeated-measures ANOVA when the assumption of normality was satisfied. If the sphericity assumption was violated, the Greenhouse-Geisser correction was applied. For non-parametric data, Friedman’s ANOVA was used. Cohen’s effect sizes were calculated to determine the magnitude of the changes over time. Reliability of the measured variables was assessed using Cronbach’s α coefficient and the 95% CI of the mean. | Thank you for this helpful comment. We have revised the Data Analysis section to explicitly report that normality was assessed using the Kolmogorov–Smirnov test. We also clarified that repeated-measures ANOVA was used for variables meeting the normality assumption, whereas Friedman’s ANOVA was applied when the assumption was violated.:  The collected data were analyzed using SPSS for Windows (version 26.0; SPSS Inc., Chicago, IL, USA). Normality of the main outcome variables was assessed using the Kolmogorov–Smirnov test prior to inferential analysis. The general characteristics of the participants were analyzed using frequencies, percentages, means, and standard deviations. Variables that satisfied the normality assumption, including basic and advanced clinical knowledge and self-efficacy, were analyzed using repeated-measures analysis of variance (ANOVA). If the sphericity assumption was violated, the Greenhouse-Geisser correction was applied. Clinical performance ability variables did not meet the normality assumption and were therefore analyzed using Friedman’s ANOVA. Cohen’s effect sizes were calculated to determine the magnitude of the changes over time. Reliability of the measured variables was assessed using Cronbach’s α coefficient and the 95% CI of the mean. |
| Reduce minor redundancy in the Discussion and perform a final language polish. | This study developed and implemented an SCCDP to improve the clinical performance of new nurses, support their effective adaptation to clinical settings, and increase their intention to remain in the workplace. To assess its effectiveness, the SCCDP was structured into basic and advanced stages and applied over time. Clinical knowledge, clinical performance ability, and self-efficacy were evaluated to confirm the program’s effectiveness. Verification of the effectiveness of the SCCDP revealed improvements in both basic and advanced clinical knowledge and clinical performance ability following training. Notably, the SCCDP demonstrated a lasting impact, with effects sustained immediately after implementation and continuing at three and six months post-training. These results highlight that structured, longitudinal competency-based education can effectively bridge the gap between initial orientation and ongoing professional development, providing sustained support for new nurses beyond the early transition phase.  …  Education led by clinical nurse educators has been positively evaluated before, as it enables systematic and level-specific instructions, which has been proven to reduce anxiety among new nurses [11, 29]. The amateur nursing skills of new nurses, challenges stemming from the gap between theoretical content and practice, and pressure to perform well contribute to psychological anxiety [30]. Furthermore, improper education that does not teach the appropriate skill levels may widen this gap. To address this, retention rates may be improved by providing training that combines practice and simulations tailored to the developmental and adaptation stages of new nurses [16]. In addition, the gap between the theoretical knowledge required at each stage and practical applications in clinical settings can be minimized, and simulation practice based on real cases can enhance learning confidence while reinforcing critical thinking skills [31]. This aligns with meta-analytic evidence indicating that simulation-based education is particularly effective when aligned with learners’ developmental needs [32]. Our results further support the need for stepwise education for new nurses engaged in intensive work, as suggested by prior Korean and international studies [9, 29, 33]. In particular, the sustained improvement in medical device operation and emergency nursing competencies emphasizes that structured repetition and simulation contribute to safer clinical practice. To achieve broader impact, future applications of the SCCDP should be tested in varied hospital settings and compared with existing residency programs to evaluate scalability and standardization. | Thank you for this comment. Minor redundancy in the Discussion section was reduced by consolidating overlapping statements, and a final language polish was performed to improve clarity and conciseness  while preserving the original structure and interpretation.:  This study developed and implemented an SCCDP to improve the clinical performance of new nurses, support their effective adaptation to clinical settings, and increase their intention to remain in the workplace. To assess its effectiveness, the SCCDP was structured into basic and advanced stages and applied over time. Clinical knowledge, clinical performance ability, and self-efficacy were evaluated to confirm the program’s effectiveness. The findings demonstrated significant improvements in both basic and advanced clinical knowledge and clinical performance ability following program participation. Importantly, these effects were sustained immediately after the program and at three- and six-month follow-up assessments. These findings suggest that structured, longitudinal competency-based education can effectively bridge the gap between initial orientation and ongoing professional development for new nurses.  ….  Education led by clinical nurse educators has been positively evaluated before, as it enables systematic and level-specific instructions, which has been proven to reduce anxiety among new nurses [11, 29]. New nurses often experience psychological anxiety due to limited practical skills, gaps between theoretical knowledge and clinical practice, and pressure to perform competently [30]. Furthermore, improper education that does not teach the appropriate skill levels may widen this gap. To address this, retention rates may be improved by providing training that combines practice and simulations tailored to the developmental and adaptation stages of new nurses [16]. In addition, the gap between the theoretical knowledge required at each stage and practical applications in clinical settings can be minimized, and simulation practice based on real cases can enhance learning confidence while reinforcing critical thinking skills [31]. This aligns with meta-analytic evidence indicating that simulation-based education is particularly effective when aligned with learners’ developmental needs [32]. Our results further support the need for stepwise education for new nurses engaged in intensive work, as suggested by prior Korean and international studies [9, 29, 33]. In particular, the sustained improvement in medical device operation and emergency nursing competencies emphasizes that structured repetition and simulation contribute to safer clinical practice. To achieve broader impact, future applications of the SCCDP should be tested in varied hospital settings and compared with existing residency programs to evaluate scalability and standardization. |
| Ensure consistency in terminology and formatting throughout the manuscript. | Terminology and formatting were not fully standardized across the manuscript. | Thank you for this comment. We carefully reviewed the entire manuscript to ensure consistency in terminology and formatting. Key terms and abbreviations were standardized throughout the text, tables, and figures. Specifically, the term “new nurses” was used consistently to refer to the study population, and the program name was standardized as “Stepwise Clinical Competency Development Program (SCCDP)” after its initial definition. |
